# Reproductive biology and conservation status of *Leopoldia mira*, *Muscari sandrasicum* and *M. serpentinicum* (Asparagaceae)

**DOI:** 10.3897/BDJ.13.e159983

**Published:** 2025-09-05

**Authors:** Okan Çon, Mehmet Çiçek

**Affiliations:** 1 Pamukkale University, Faculty of Science, Department of Biology, Denizli, Turkiye Pamukkale University, Faculty of Science, Department of Biology Denizli Turkiye; 2 Pamukkale University, Faculty of Science, Department of Molecular Biology and Genetics, Denizli, Turkiye Pamukkale University, Faculty of Science, Department of Molecular Biology and Genetics Denizli Turkiye

**Keywords:** IUCN, population, reproductive success, threat category

## Abstract

*Leopoldia mira*, *Muscari sandrasicum* and *M. serpentinicum* are endemic grape hyacinth species restricted to serpentinous and calcareous habitats in south-western Anatolia, Türkiye. Their pollination types, pollen and seed viability and primary pollinators were investigated through population observations, pollination experiments and various tests. A mixed pollination system was found to be predominant in *L. mira* and *M. serpentinicum*, whereas *M. sandrasicum* exhibited obligate xenogamy (outcrossing). Additionally, all species were observed to be entomophilous. New populations of *L. mira* and *M. sandrasicum* were discovered on Eşeler Mountain (Denizli, Türkiye). Their IUCN threat categories were re-assessed using the new data obtained in this study. The threat categories of *L. mira* and *M. sandrasicum*, previously classified as Endangered (EN), were retained, while *M. serpentinicum* should be reclassified as EN, based on criterion B2ab(ii,iii,iv,v)c(iii) due to its limited population size and increasing threats such as grazing, habitat degradation and climate change.

## Introduction

Habitat degradation and major climatic changes can lead to a reduction in the area of occupancy of species and even their extinction; however, reproductive success is crucial for their survival. Genetic variations within and amongst populations, as well as population dynamics, are directly related to the reproductive capacity of species (Kwak and Bekker 2006, Corlett 2007). Understanding reproductive biology is essential for developing effective conservation strategies for endemic and threatened species (Fenner and Thompson 2005, Barrett 2010). Reproductive success and pollination type influence a species' regenerative capacity, genetic diversity and adaptability to environmental changes (Kearns and Inouye 1993, Harder and Barrett 2006). Additionally, this information helps to identify potential causes of reproductive failure, population decline or restricted distribution.

The IUCN Red List Categories and Criteria are primarily used to assess the global extinction risk of threatened species and to determine their threat status (IUCN 2024). Robust data on population size, geographic range, habitat quality and known threats — many of which are directly related to reproductive success and demography —are crucial for accurate categorisation, based on the IUCN system (Akçakaya et al. 2000, Mace et al. 2008). Integrating reproductive biology data with IUCN Red List Criteria is especially important for conservation assessments in biodiversity-rich regions, such as the Mediterranean Basin, which face habitat fragmentation and human pressures (Médail and Quézel 1999, Myers et al. 2000). Such studies can provide information for habitat management, ex situ conservation efforts and species recovery plans by identifying reproductive constraints and potential vulnerabilities (Briskie and Mackintosh 2004, Havens et al. 2004).

The genus *Muscari* Miller, which comprises 46 species worldwide, has a distribution that extends from the Mediterranean Basin, including southern Europe, to Central Asia (including the Caucasus Region). The centre of diversity for this genus is considered to be Anatolia, which hosts two-thirds of its species (Eker 2022). *Leopoldia* Parlatore is represented globally by 29 species distributed across the Mediterranean Basin, the Middle East, central and southern Europe, the Caucasus and south-western Asia. Western Anatolia and the Aegean Islands, where more than half of *Leopoldia* species naturally occur, are recognised as diversity hotspots for the genus (Eker 2022). Recent morphological and molecular studies suggest that certain species, such as *L. mira* (= *M. mirum*), occupy a distinctly separate position from other members of the subgenus Leopoldia. Dizkirici A et al. (2018) reported that *L. mira* (= *M. mirum*) differs notably from related taxa in morphology, including a shorter stature, broader leaves and larger fruits and emphasised the need for further studies to clarify its phylogenetic placement. However, Leopoldia has been treated as a subgenus nested within a broadly defined *Muscari* in a recent phylogenetic study (Böhnert T et al. 2023). In this study, we have adopted *Leopoldia* at the generic rank (Eker 2022).

**Figure 1.**
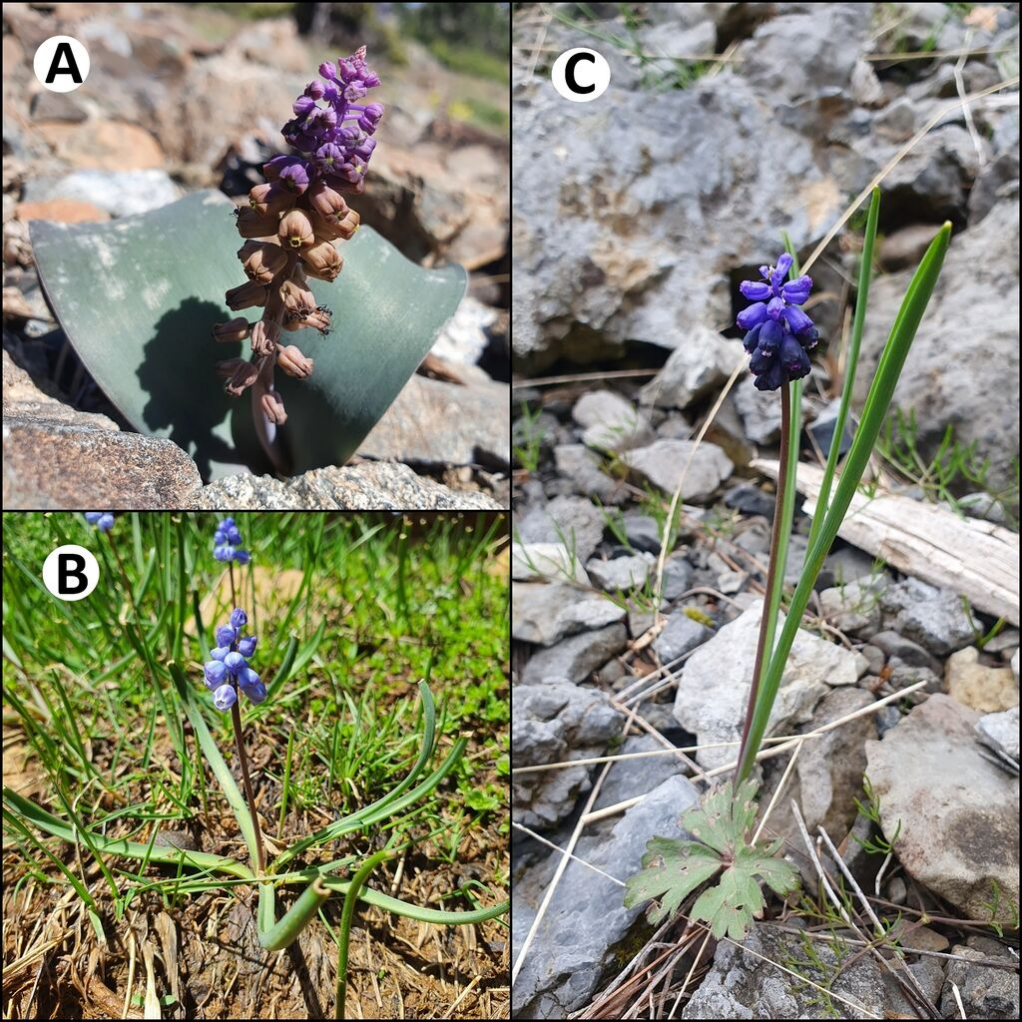
*Leopoldia mira* (**A**), *Muscari sandrasicum* (**B**) and *M. serpentinicum* (**C**) in their natural habitats.

*Leopoldia mira*, *Muscari sandrasicum* and *Muscari serpentinicum*, known from restricted populations, face significant threats, such as habitat degradation and anthropogenic impacts. Previous studies have classified *L. mira* and *M. sandrasicum* as “Endangered (EN)” (Ekim et al. 2000) and *M. serpentinicum* as “Vulnerable (VU)” (Pirhan et al. 2014). To the best of our knowledge, except for a study on the genetic diversity, conservation strategy and population dynamics of *Muscari adilii* — a rare species occurring in Türkiye (Keser et al. 2023), no literature data are available on the reproductive biology and conservation of *Leopoldia* and *Muscari* species in Türkiye. This study focuses on the reproductive biology and re-evaluation of the threat status of three endemic grape hyacinth species — *L. mira*, *M. sandrasicum* and *M. serpentinicum* — narrowly distributed in south-western Anatolia (Türkiye) (Fig. 1). Furthermore, it aims to determine their pollination types, pollen stainability, seed viability, identify their main pollinators and assess the biotic and abiotic threats to their survival.

## Material and methods

### The spatial distribution and census of the populations

The populations of *L. mira*, *M. sandrasicum* and *M. serpentinicum* are found in the Provinces of Denizli, Muğla, Burdur and Kütahya (Table 1). All known populations were documented through field studies conducted between 2022 and 2024. To estimate the number of mature individuals — defined as those capable of reproduction, exhibiting developed flowers or seeds — different methods were applied depending on the size of the population’s habitat. For populations occupying an area smaller than 1 km², mature individuals were counted directly. For larger populations, ten 25 m² quadrats were randomly selected from various parts of the population area. The number of mature individuals within each quadrat was recorded and the average density was used to estimate the total number of mature individuals across the entire habitat. Google Earth was utilised to calculate the total surface area occupied by each population. For conservation status assessment, the IUCN (International Union for Conservation of Nature) Criteria were applied, specifically using the 2 x 2 km grid approach. This grid was employed via the GeoCAT tool to calculate the Area of Occupancy (AOO) and Extent of Occurrence (EOO). The grid typically incorporates parameters, such as the extent of distribution (EOO) and the number of occupied locations (AOO or number of localities), which are particularly relevant for assessments under Criterion B, focusing on geographic range.

### Mode of pollination

To determine the pollination types of *L. mira*, *M. sandrasicum* and *M. serpentinicum*, experimental and observational pollination studies were conducted in their natural populations throughout the flowering periods of 2023 and 2024.

#### Direct method for pollination experiments

**Figure 2.**
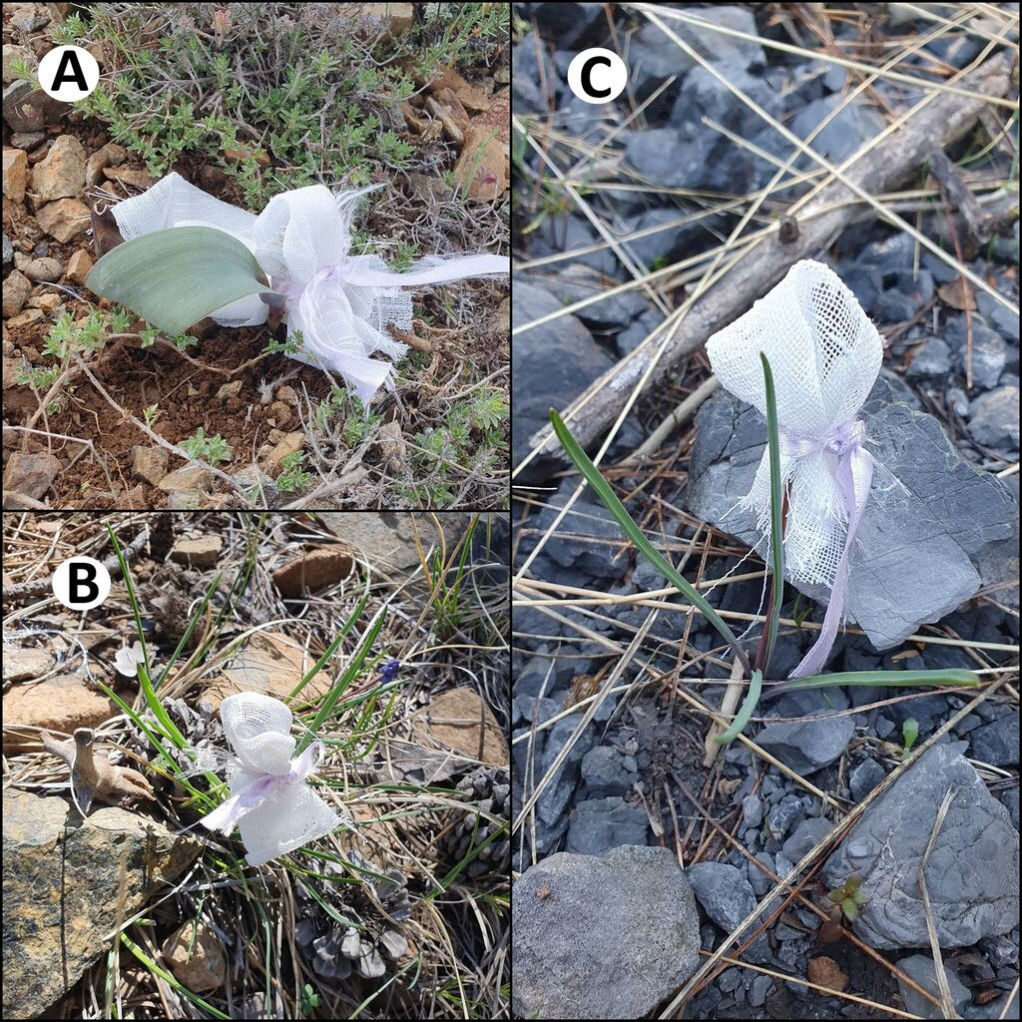
Pollination experiments of *Leopoldia mira* (**A**), *Muscari sandrasicum* (**B**) and *M. serpentinicum* (**C**) conducted in their natural habitats.

Three different treatments were applied in the pollination experiments. For each treatment, populations representing the largest habitat area of each taxon were selected to best reflect their reproductive potential under natural conditions. To test for self-pollination (autogamy), 10 individuals at the bud stage were randomly selected from each population and isolated using cheesecloth bags to prevent insect visitation throughout the flowering period. For wind pollination, the anthers of fertile flowers from 10 randomly selected individuals were carefully removed and the plants were then re-isolated with cheesecloth. This isolation step was essential to ensure that only wind-mediated pollination occurred, as the presence of insects could lead to unintended biotic pollination and result in misleading data. Additionally, since the pollen grains of the studied species range between 35 and 45 µm in diameter and can easily pass through the mesh openings of approximately 1 mm² in the cheesecloth, wind pollination remained possible despite the physical barrier. For natural pollination (control group), 10 randomly selected individuals were marked with red ribbons and left under natural conditions, allowing both insect and wind pollination. All experimental treatments were monitored until the end of the fruiting period and fruit set was recorded (Fig. 2).

#### Indirect method to determine pollen/ovule (P/O) ratios

In calculating pollen-to-ovule (P/O) ratios, unopened fertile flowers at the bud stage were used. For each species, individuals were selected from populations with the largest habitat areas to best represent the natural reproductive potential of the taxa. Amongst these, the most representative and healthy individuals were chosen, based on morphological integrity and flower number. Five individuals per species were selected and five unopened flowers were collected from each individual, resulting in a total of 25 flowers.

To estimate pollen production, one anther was randomly selected from each flower. A slide was prepared for each anther by crushing it in a drop of water and covering it with a coverslip. Pollen grains were counted under a light microscope at 100× magnification using a zigzag scanning pattern across the entire slide. In total, 25 anthers were examined per species and the average number of pollen grains per anther was calculated.

For ovule counts, the ovaries of 25 flowers per species were carefully dissected under a stereomicroscope and the number of ovules in each flower was recorded to calculate the average ovule number. The pollen-to-ovule (P/O) ratios were then calculated by dividing the mean number of pollen grains per anther by the mean number of ovules per flower. The results were interpreted according to the Cruden (1977) classification of reproductive strategies.

### Pollinators

Insect visitors to the populations of *Leopoldia mira*, *Muscari sandrasicum* and *M. serpentinicum* were observed and photographed during their respective flowering periods in 2023 and 2024. Pollinator observations were conducted throughout the blooming phases of each species: in May for *L. mira* and *M. sandrasicum* and in April for *M. serpentinicum*.

### Pollen viability

During the pollination period, flower samples were collected and placed in small bottles filled with water, then transported to the laboratory in a portable freezer to preserve their viability. For assessing pollen viability, a TTC (2,3,5-Triphenyl-Tetrazolium Chloride) solution was dripped on to a slide and pollen grains were gently transferred on to the drop using a small brush. The slide was then covered with a coverslip and stained for 2 hours. Pollen grains stained red were considered viable and counted under a light microscope (Norton 1966, Eti 1991). For each location, five individuals were sampled and one slide was prepared per individual. A total of 100 pollen grains were counted per location to determine the proportion of viable and non-viable pollen. To calculate species-level viability ratios, the average values from all locations corresponding to the same species were used.

### Seed viability

Mature seeds of *L. mira*, *M. sandrasicum* and *M. serpentinicum* were randomly collected from their natural populations and stored in a cool, dry and dark environment for analysis. Seed viability was assessed using Triphenyl Tetrazolium Chloride (TTC) staining (Moore 1985). For each species, 20 mature seeds were randomly selected and soaked in distilled water for 24 hours to soften the seed coat and facilitate embryo extraction. After soaking, the seed coats were carefully peeled and the extracted embryos were immersed in a 1% TTC solution and incubated in the dark at room temperature (approximately 22–25°C) for 24 hours. Following the TTC procedure, the embryos were examined under a stereomicroscope; embryos staining red were considered viable, while those unstained were deemed non-viable.

### Conservation status

AOO (Area of Occupancy) and EOO (Extent of Occurrence) values for the species were calculated using the IUCN mapping tool GeoCAT (Geospatial Conservation Assessment Tool, https://geocat.iucnredlist.org/). According to the IUCN (2024) Criteria, the threat categories of all species were re-assessed by considering the AOO and EOO values, the number of mature individuals, the number of locations and the primary threats.

## Results

### The spatial distribution and census of the populations

Three populations of *L. mira* were identified at Dirmil, Aliveren and Eşeler Mountain. Its area of occupancy was calculated to be 6.9 km², with a total of 17600 individuals counted. *Muscari sandrasicum* was represented by three populations — Sandras Mountain, Bozdağ and Eşeler Mountain — comprising a total of 40164 individuals and its area of occupancy was calculated as 18.802 km². A total of 14505 individuals of *M. serpentinicum* were recorded across eight populations located at Şaphane Mountain, Çakmak, Koru Plateau, Karanfilli Plateau, Aliveren, Milas, Sandras Mountain and Yılanlı Mountain, with an area of occupancy calculated at 1.6 km² (Table 2).

### Pollination types

Fruit set was evaluated in three species under both bagged (to exclude pollinators) and non-bagged (control) conditions. In *Muscari serpentinicum*, fruit developed in 29 of 108 fertile flowers (26.85%) from 10 randomly selected bagged individuals. In contrast, the control group (non-bagged) exhibited a higher fruit set, with 65 of 126 fertile flowers (51.58%) producing fruit. In *M. sandrasicum*, no fruit set was observed amongst the 87 fertile flowers in the bagged group, whereas 62 of 97 fertile flowers (63.91%) in the control group successfully set fruit. For *Leopoldia mira*, fruit set occurred in 105 of 217 fertile flowers (48.38%) in the bagged group and in 187 of 263 flowers (71.1%) in the control group (Table 3). In wind-pollination experiments, no fruit development was observed in any emasculated (anther-removed) and bagged flowers across all species, indicating that wind does not play a significant role in pollination.

Average pollen production per anther was calculated as 5287 pollen grains in *L. mira*, 10228 in *M. sandrasicum* and 6220 in *M. serpentinicum*. Considering that each flower contains six stamens, the average pollen production per flower was estimated at 31722, 61368 and 37320 pollen grains, respectively. Ovule numbers ranged from 6 - 18 amongst the studied species, with an average of 12 ovules per flower used in calculations. Based on these values, the pollen-to-ovule (P/O) ratios were determined as 2643.5 (log 3.42) in *L. mira*, 5114 (log 3.7) in *M. sandrasicum* and 3110 (log 3.49) in *M. serpentinicum*. According to Cruden’s (1977) classification, these P/O ratios indicate a mixed pollination system in *L. mira* and *M. serpentinicum* and an obligate xenogamous (outcrossing) reproductive strategy in *M. sandrasicum* (Table 4).

### Pollinators

**Figure 3.**
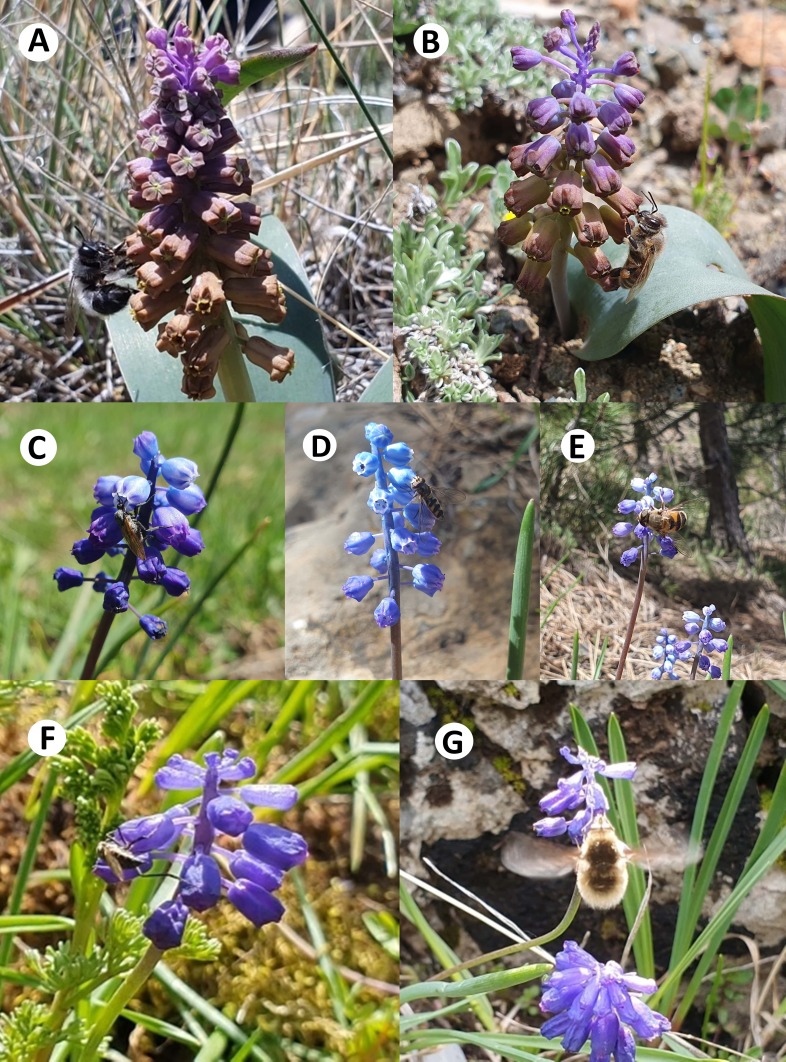
The pollinators of *Leopoldia mira* (**A**: *Bombus* sp., **B**: *Apis mellifera*), *Muscari sandrasicum* (**C**: *Empis* sp., **D**: *Meliscaeva* sp., **E**: *Apis mellifera*) and *Muscari serpentinicum* (**F**: *Empis* sp., **G**: *Bombus* sp.).

In the pollinator monitoring studies, we observed *Apis mellifera* to be the primary pollinator for all species studied. Additionally, *Bombus* sp. was the secondary pollinator for *L. mira* and *M. serpentinicum*; *Empis* sp. for *M. sandrasicum* and *M. serpentinicum*; and *Meliscaeva* sp. for *M. sandrasicum* (Fig. 3).

### Pollen viability

A total of 400 pollen grains were evaluated across four sub-populations of *Leopoldia mira*. The highest overall pollen viability rate was observed in *L. mira*, with a mean of 85.5%. At the population level, the Aliveren population exhibited the highest viability at 92%, followed by Dirmil at 87%, Eşeler at 82% and two other sub-populations at 81%. In *Muscari sandrasicum*, 600 pollen grains were assessed from six sub-populations, yielding an overall viability rate of 81.5%. The pollen viability rates for the respective populations were as follows: Sandras Mountain (four sub-populations) at 72%, 81%, 88% and 90%; Bozdağ at 87%; and Eşeler at 71%. For *M. serpentinicum*, 900 pollen grains were counted from nine sub-populations, with an average viability rate of 74.33%. Population-specific viability rates were: Şaphane 86%, Çakmak 84%, Koru Plateau 78%, Karanfilli Plateau 74%, Aliveren 59%, Milas 62%, Sandras Mountain (two sub-populations) 68% and 76% and Yılanlı Mountain 82%.

### Seed viability

When stained with TTC solution, 40% of *L. mira* seeds stained red or dark red, while 60% did not stain. For *M. sandrasicum*, 60% of the seeds stained and 40% did not. In the case of *M. serpentinicum*, 80% of the seeds stained and 20% did not. Based on these results, the seed viability was determined to be 40% for *L. mira*, 60% for *M. sandrasicum* and 80% for *M. serpentinicum*.

## Discussion

The reproductive biology of *Leopoldia mira*, *Muscari sandrasicum* and *M. serpentinicum* was investigated, based on pollen-to-ovule (P/O) ratios and fruit set data obtained from controlled pollination experiments. The results revealed distinct differences in their reproductive strategies. While *L. mira* and *M. serpentinicum* were capable of producing seeds through self-pollination to some extent, *M. sandrasicum* was largely dependent on pollinators for seed production. Cruden’s (1977) classification of reproductive strategies, based on P/O ratios, highlights clear differences amongst the studied species. *Leopoldia mira* and *Muscari serpentinicum* appear to follow a mixed pollination system, suggesting that both self- and cross-pollination contribute to their reproductive success. In contrast, *Muscari sandrasicum* is characterised by obligate xenogamy, indicating a strict dependence on pollinators for successful seed set. These differences may be related to species-specific floral traits, pollinator interactions or ecological factors shaping their reproductive strategies. The fruit set results further support these observations. In *M. sandrasicum*, none of the 87 isolated flowers (with pollinator access excluded) set fruit, indicating self-incompatibility and a requirement for pollinators. Our findings align with those of Herrmann N et al. (2006), who characterised *Leopoldia tenuiflora* (reported as *Muscari tenuiflorum* in their study) as facultatively xenogamous, based on its pollen-to-ovule ratio and fruit set under different pollination treatments. Similar to their observations, *L. mira* and *M. serpentinicum* in our study demonstrated fruit set in both isolated and open-pollinated flowers, indicating a capacity for self-pollination, but enhanced reproductive success in the presence of pollinators. This suggests a mixed mating system in which cross-pollination predominates, but self-pollination can occur in the absence of pollinators. In contrast, *M. sandrasicum* showed no fruit set in bagged flowers, confirming obligate xenogamy and complete reliance on insect pollinators for reproduction. These results are also supported by P/O ratios, further validating the classification of the reproductive systems observed. Moreover, the absence of fruit set in wind-pollination treatments across all species confirms that effective pollination is mediated exclusively by insects, reinforcing the entomophilous nature of these taxa.

In our pollinator monitoring studies, *Apis mellifera* was identified as the primary pollinator for all the species examined, while *Bombus* sp. (for *L. mira* and *M. serpentinicum*), *Empis* sp. (for *M. sandrasicum* and *M. serpentinicum*) and *Meliscaeva* sp. (for *M. sandrasicum*) were recorded as secondary pollinators. These findings indicate a relatively diverse pollinator assemblage, involving both social bees and dipteran species. In comparison, Canale A et al. (2013) reported that the pollinator spectrum of *Leopoldia comosa* (referred to as *Muscari comosum* in their study) was dominated by generalist solitary bees, particularly *Anthophora plumipes* and *Halictus tarsata*, which accounted for 85.72% of all visits, while social bees, such as *A. mellifera* and *Bombus terrestris*, comprised 14.28% of the visitors. They also noted the presence of kleptoparasitic species (Melecta albifrons var. nigra and *Nomada sexfasciata*), which were observed collecting nectar, but not pollen and, thus, did not contribute to effective pollination. Compared to their findings, our results highlight a greater role of *A. mellifera* as a consistent and dominant pollinator, alongside the involvement of a broader range of taxa, particularly dipterans, in the species we studied. This suggests interspecific differences in pollination ecology even amongst closely-related taxa and emphasises the importance of both taxonomic and functional diversity amongst floral visitors.

Pollen and seed viability are important indicators of reproductive success (Dafni and Maués 1998). The pollen viability rates were determined to be 87% for *L. mira*, 81.5% for *M. sandrasicum* and 74.3% for *M. serpentinicum*. High pollen viability primarily results from regular meiosis during pollen development, with environmental factors having only a minor effect. Significant differences were observed in seed viability amongst the species: *L. mira* exhibited a low viability rate of 40%, while *M. sandrasicum* and *M. serpentinicum* showed higher rates of 60% and 80%, respectively. These differences can be attributed not only to fertilisation success, but also to seed development, maturation processes, environmental stress factors and genetic structure. Notably, in *L. mira*, despite high pollen viability (87%), seed viability was relatively low (40%), suggesting a disruption in the developmental process after fertilisation. This may be due to physiological disorders during embryo development, unfavourable environmental conditions (such as temperature, humidity and light) or genetic factors.

In *M. serpentinicum*, pollen and seed viability, as well as reproductive success, were higher than in the other two species. The high percentage of viable seeds is primarily attributed to intrinsic seed traits, such as robust embryo development and resistance to environmental stresses.

Pollen viability influences the success of fertilisation, while seed viability directly impacts the formation of a new generation. Therefore, seed viability is crucial for reproductive success, even when pollen viability is high. Populations with high reproductive success are expected to exhibit both high pollen viability and high seed viability.

In conservation studies, understanding the population dynamics, reproductive biology and life cycle of a species is essential for the long-term persistence of populations and the development of effective conservation strategies (Ohara et al. 2006). In particular, unless the factors threatening their survival are identified and mitigated, threatened species are likely to face extinction in the near future. Prior to this study, *L. mira* was known from two populations located in Dirmil (Burdur) and Aliveren (Denizli) (Speta 1989, Eroğlu 2020, Eker 2022). *M. sandrasicum* was known from two populations: one in Sandras Mountain (Denizli and Muğla) and the other in Bozdağ (Denizli). *M. serpentinicum* was recorded from a total of eight populations distributed across the Provinces of Kütahya, Denizli and Muğla. We discovered new populations of *L. mira* and *M. sandrasicum* on Eşeler Mountain (Denizli, Acıpayam). With these new discoveries, *L. mira* is currently represented by 17600 individuals across a total area of 6.9 km² and *M. sandrasicum* by 40164 individuals across 18.802 km². Additionally, we confirmed the presence of eight populations of *M. serpentinicum* by counting 14505 individuals over a total area of 1.6 km² during visits to previously recorded sites.

The threat status of *L. mira* was initially assessed as Endangered (EN) in the Red Book of Plants of Türkiye (Ekim et al. 2000) and later confirmed by Eroğlu (2020), based on criterion B1a,c(i). Based on our new data regarding location, as well as Area of Occupancy (AOO) and Extent of Occurrence (EOO) values, we propose that it should remain classified as Endangered under criteria B1+B2ab(ii,iii,v)c(ii,iv) (IUCN 2024). The primary threat to this species is grazing by small ruminants, which exploit its habitat depending on its use as grazing land. Additionally, terracing, tree planting and the proximity of its habitats to human settlements pose significant threats to *L. mira* populations.

*Muscari sandrasicum* was previously classified as Endangered (EN) by earlier authors (Ekim et al. 2000, Eroğlu 2020). Based on the IUCN (2024) Criteria and our recent field observations, we confirm the Endangered status for *M. sandrasicum*, supported by new data on the number of locations, as well as Area of Occupancy (AOO) and Extent of Occurrence (EOO) values, which meet the B1+B2ab(ii,iii)c(ii,iv) criteria. However, we anticipate that the main population of *M. sandrasicum* may face increased threats in the near future, as its immediate habitat is becoming vulnerable to human activities, such as camping, picnicking, grazing and skiing.

*Muscari serpentinicum* was initially described as a species with a very narrow distribution, represented by only 270 individuals and classified as Vulnerable (VU) according to the D1 criterion (Pirhan et al. 2014). Eroğlu (2020) also confirmed the VU category, based on criteria a and c(i). However, due to the species being represented by even smaller populations and a reduced number of individuals within these populations and considering the imminent risk of extinction, we propose reclassifying *M. serpentinicum* as Endangered (EN), based on criterion B2ab(ii,iii,iv,v)c(iii). In addition to the low population size, the primary threat to *M. serpentinicum* is habitat degradation caused by overgrazing.

For this species, the extreme fluctuations in their populations can be largely attributed to habitat degradation caused by the intensity of agricultural and forestry activities within their natural distribution ranges, reduced snowfall and shorter snow cover duration due to climate change. These factors disrupt dormancy processes, leading to delayed germination.

## Conclusions

As a result of this study, the threat category for the species studied herein is proposed as Endangered (EN). The primary threat is intense grazing pressure throughout their range. We recommend monitoring their population for at least 10 years, protecting their habitat, storing their seeds in seed banks and conducting ex-situ and/or in-situ conservation studies to ensure the survival of these species.

## Figures and Tables

**Table 1.** Geographic distribution and elevation data of the species.

**Species**	**Locality Name**	**Province**	**Coordinates**	**Elevation (m)**
*Leopoldia mira*	Dirmil	Burdur	36°58'3"N, 29°34'45"E	1250-1700
*Leopoldia mira*	Aliveren	Denizli	37°12'50"N, 29°26'19"E	1200-1550
*Leopoldia mira*	Eşeler Mountain	Denizli	37°20'39,7"N, 29°36'5,8"E; 37°30'39,8"N, 29°40'13,2"E	1250-1830
*Muscari sandrasicum*	Sandras Mountain	Denizli-Muğla	37°05'44"N, 28°50'49"E; 37°03'37"N, 28°48'19"E; 37°03'17"N, 28°47'42"E; 37°5'35"N, 28°53'23"E	1350-2100
*Muscari sandrasicum*	Bozdağ Mountain	Denizli	37°18'37"N, 29°11'10"E	1600-2300
*Muscari sandrasicum*	Eşeler Mountain	Denizli	37°24'14"N, 29°38'30"E	1650-1750
*Muscari serpentinicum*	Şaphane Mountain	Kütahya	39°02'22"N, 29°17'21"E	1800-2050
*Muscari serpentinicum*	Çakmak	Muğla	37°11'49"N, 28°39'07"E	830
*Muscari serpentinicum*	Koru Plateau	Muğla	36°50'18"N, 29°11'47"E	1160
*Muscari serpentinicum*	Karanfilli Plateau	Muğla	36°52'04"N, 29°12'17"E	1500
*Muscari serpentinicum*	Aliveren	Denizli	37°13'31"N, 29°26'46"E	1300-1400
*Muscari serpentinicum*	Milas	Muğla	37°18'2,2"N, 27°56'54,7"E	690
*Muscari serpentinicum*	Sandras Mountain	Denizli- Muğla	37°5'37,7"N, 28°53'16"E; 37°2'31,6"N, 28°46'50,4"E	1400-1550
*Muscari serpentinicum*	Yılanlı Mountain	Muğla	37°13'56,7"N, 28°39'13,8"E	1300

**Table 2.** AOO and EOO values and the total number of mature individuals in the populations.

Species	Extent of occurrence EOO (km^2^)	Area of occupancy AOO (km^2^)	The total number of mature individuals	Total surface area covered by populations (km^2^)
*Leopoldia mira*	875	50	17600	6.9
*Muscari sandrasicum*	772	48	40164	18.802
*Muscari serpentinicum*	16439	40	14505	1.6

**Table 3.** Fruit set and flowering data under various pollination treatments.

**Species**	**Isolated flowers-Fruit Count**	**Isolated flowers-Flower Count**	**Isolated flowers-Fruit Set Rate (%)**	**Control-Fruit Count**	**Control-Flower Count**	**Control-Fruit Set Rate (%)**	**Wind Pollination-Fruit**
*Leopoldia mira*	105	217	48.38	187	263	71.1	No fruit set
*Muscari sandrasicum*	0	87	0	62	97	63.91	No fruit set
*Muscari serpentinicum*	29	108	26.85	65	126	51.58	No fruit set

**Table 4.** Floral traits and reproductive system classification, based on pollen-ovule ratios.

Species	Pollen per Anther	Stamens per Flower	Total Pollen per Flower	Average Ovules per Flower	P/O Ratio	log₁₀ (P/O)	Reproductive System
*Leopoldia mira*	5287	6	31722	12	2643.5	3.42	Mixed mating system
*Muscari sandrasicum*	10228	6	61368	12	5114	3.7	Obligate xenogamy (outcrossing)
*Muscari serpentinicum*	6220	6	37320	12	3110	3.49	Mixed mating system
